# Green biogenic sulfur nanoparticles enhance *Capsicum annuum* (L.) resilience to salt stress by triggering physio-biochemical and genetic repair mechanisms

**DOI:** 10.3389/fpls.2025.1564621

**Published:** 2025-03-07

**Authors:** Hissah Alrabie, Hameed Alsamadany, Ameina S. Almoshadak, Rahma Alshamrani, Manal El-Zohri

**Affiliations:** ^1^ Department of Biological Sciences, Faculty of Science, King Abdulaziz University, Jeddah, Saudi Arabia; ^2^ Department of Botany and Microbiology, Faculty of Science, Assiut University, Assiut, Egypt

**Keywords:** green synthesis, sulfur nanoparticles, moringa; sweet pepper, salinity, water relations, antioxidant system, salt-tolerant genes

## Abstract

The synthesis of metal nanoparticles is an expanding field of study due to the potential uses in creating new technologies that facilitate the production of crops by improving tolerance against salinity stress. The current study outlined the green synthesis of sulfur nanoparticles (SNPs) using *Moringa oleifera* (Lam.) leaf extract and its protective role on *Capsicum annuum* (L.) growth against salinity stress. Using Fourier transform infrared (FT-IR) spectroscopy, transmission electron microscopy (TEM), and X-ray diffraction (XRD), the effective formation of the synthesized SNPs was examined and approved. The results confirmed the purity and morphology of SNPs. Then, SNPs (1, 10, 100 mg/l) were used in nano-priming to alleviate the adverse effects of NaCl (50, 100 mM) on *C. annuum* seedlings. The findings demonstrated that *C. annuum* growth parameters were severely lowered by increasing salinity stress level, whereas SNPs treatments enhanced plant growth under both salt levels. The optimum concentration for alleviating salinity stress was 10 mg/l SNPs. 10 mg/l SNPs significantly increased shoot fresh weight, dry weight, chlorophyll content, cell membrane stability and relative water content by 75.4, 77.8, 82.5, 89.5 and 20.9%, while reduced the water and solute potential, Na^+^/K^+^ ratio, proline, glycine betaine, malondialdehyde, H_2_O_2_ and superoxide anion content by 45.5, 43.2, 27.7%, 18.1, 40.3, 39.3, 35.4 and 34.5% respectively compared to untreated stressed control at 100 mM NaCl. Moreover, SNPs substantially improved, antioxidant enzymes activities and upregulated the expression of some salt-tolerant genes under saline conditions. Under both salinity levels, the genes *CaHAK6*, *CaHAK7*, *CaDHN3*, *CaCAT1* and *CaPOD* recorded maximum expression at 10 mg/l SNPs. Overall, these findings demonstrate the efficiency of green SNPs as a practical approach to alleviate NaCl-induced stress in *C. annuum* plants by triggering many physiological, biochemical and genetic repair mechanisms. These results offer a sustainable agri-environmental strategy for mitigating salt stress and enhancing crop production in saline environments.

## 1 Introduction

According to the Food and Agriculture Organization (FAO), around 20% of the agricultural farmland is affected by salinity, which is predicted to considerably increase in the coming decades as a consequence of industrialization, over fertilization, increased use of low quality irrigation water, soil salinization, and natural causes such as salt intrusion in coastal zones due to rising sea levels (Anwar et al., 2022). Therefore, salinity represents a significant challenge for agriculture worldwide, mainly about 90% of plant-based food coming from the thirty most salt-sensitive crops (Shelar et al., 2024). There are several studies reported that majority of the cultivated lands in Saudi Arabia (SA) are affected by salinity at different levels (Hussain and Alshammary, 2008; Al-Hassoun, 2009; Elhag, 2016). Furthermore, this problem is getting worse with increasing irrigation water’s salinity where the agriculture sector in SA based mainly on groundwater. The excessive pumping of freshwater reduces the water pressure and intensifies the lateral movement of seawater into freshwater aquifers increasing its salinity (Abdalla, 2016). This represents a major challenge to sustaining agricultural practices in SA.

Soil salinity hampers plant growth and development via water stress and causes cytotoxicity due to excessive uptake of ions such as sodium (Na^+^) and chloride (Cl^−^) (Suzuki et al., 2016). The accumulation of these ions in plant tissues causes many adverse problems, such as membrane damage, irregular cell division, inhibited photosynthetic pigments, proteins synthesis and enzymatic activity hence reducing crop quality and yield production (Safdar et al., 2019). The excess amount of Na^+^ and Cl^−^ ions leads also to the overproduction of the reactive oxygen species (ROS), which are highly toxic and causes oxidative stress and damages proteins, lipids, carbohydrates, and DNA (Sun et al., 2021). Potassium is one of the abundant cellular cations required for maintaining cell turgor and other enzyme activities, and its shortage in plant cells inhibits growth. Ion disorder caused by salt stress, disrupted K^+^ nutrition mechanism due to the high concentration of Na^+^. If the ratio of Na^+^/K^+^ is high, it has many deleterious effects on plants (Noohpisheh et al., 2020).

Plants tolerate salt stress through a series of mechanisms, such as Na^+^ exclusion, Na^+^/K^+^ homeostasis, hormonal synthesis and defense related gene regulation (Hussain and Alshammary, 2008; Galvan-Ampudia and Testerink, 2011; Xu et al., 2021). The binding protein (Bip) plays an important protective role by alleviating endoplasmic reticulum stress induced by misfolded proteins due to salinity stress (Howell, 2013). Overexpression of Bip genes enhances plant tolerance to environment stresses (Yang et al., 2016). Several genes have been studied in previous decades that are involved in salt tolerance such as High-Affinity Potassium Transporters (HKTs) (Gu et al., 2023). Furthermore, there are several proline-rich cell wall glycoprotein genes play an important role in regulating the accumulation of proline which contribute to osmotic adjustment and oxidative stress tolerance through proline biosynthesis (Mang et al., 2004). Dehydrins (DHNs) also play an important role by maintaining cell stabilization, and protecting macromolecules when subjected to salt stress (Meng et al., 2021).

Some strategies to alleviate the negative effects of salinity on plant growth include applying organic fertilizers and foliar application of nutrients, including sulfur, potassium, and nitrogen (El-Beltagi et al., 2023). Considered a macronutrient essential to plant growth, sulfur controls how plants react to various biotic and abiotic stressors (Ragab and Saad-Allah, 2020). It plays a significant function because it is incorporated into numerous amino acids, defensive chemicals, coenzymes, vitamins, proteins, and chlorophyll (Najafi et al., 2020). Therefore, sulfur (S) is provided as a mineral nutrient supplement in agricultural fields. On the other hand, S at the nanoscale can be effectively utilized in small amounts as sulfur nanoparticles (SNPs) to reduce costs, promote plant growth, and lessen stressful conditions (Ragab and Saad-Allah, 2020).

Recently, plant physiologists have discovered intriguing new avenues to enhance plant function under stress conditions due to nanotechnology, where earlier studies showed that nanoparticles (NPs) significantly improved plant tolerance to a variety of stresses (Behboudi et al., 2018; El-Zohri et al., 2021; Zulfiqar and Ashraf, 2021). Nanoparticles are particles that have at least one dimension less than 100 nm and have a variety of distinctive characteristics, including a high surface area to volume ratio, crystal structure, adjustable pore size, and living organisms’ cellular and molecular activity (Azameti and Imoro, 2023; Thabet and Alqudah, 2024). Synthesis of NPs is typically accomplished by costly and environmentally hazardous chemical and physical procedures that need high energy and produce dangerous byproducts (Chakraborty et al., 2022). However, green biogenic methods are simple, convenient, eco-friendly, and safe (Alrobaie et al., 2024). Recently, plant’s extracts have been used as reducing agents to synthesize biogenic NPs using a bottom-up approach to green nanotechnology (Bhandari et al., 2023). *M. oleifera* leaves contain various bioactive compounds that have reducing and stabilizing effects during the synthesis of NPs (Maheshwaran et al., 2021). The green synthesized NPs could be used in agricultural systems to increase their pliability, sustainability, and efficiency in the face of global environmental stressors (Zulfiqar and Ashraf, 2021). Nanoparticles with specific characteristics significantly affect the physiology and growth of plants under salt stress, enhancing many growth parameters and the treated plant species’ anatomical, physiological, biochemical, and molecular profiles (Sarkar and Kalita, 2023).

Sweet pepper (*Capsicum annuum* L.) is the third most significant crop in the Solanaceae family, behind potatoes and tomatoes (Ahamad et al., 2024). It is a vegetable with significant economic value on a global scale due to its organoleptic properties, nutritional benefits, and contribution to a healthy human diet (Azlan et al., 2022). Pepper is rich in bioactive substances such as vitamin C, carotenoids, flavonoids, phenols, and capsaicinoids (González-García et al., 2021). However, the pepper plant is a salt-sensitive vegetable, and every 1.0 ds/m increase in salt causes a 14% decrease in fruit yield (Abdelaal et al., 2020). Potential effects of SNPs on plant health, either positive or negative, depend on plant growth conditions, plant species, and exposure concentration and duration. In the meanwhile, the mechanisms of SNPs impacts at different concentrations, mainly under stress conditions, are not fully understood. Therefore, considering the extent of SNPs effects in plants that recorded in some previous studies (Galvan-Ampudia and Testerink, 2011; Salem et al., 2016; Najafi et al., 2020; Khalifa et al., 2024), it is interesting to investigate the responses of sweet pepper, as a sensitive plant to salinity stress, when exposed to the green synthesized SNPs. Thus, the main objective of this study was to investigate the diversified relieving roles of the greenly synthesized SNPs through exploring some multifaceted (physiological, biochemical and molecular) mechanisms by which SNPs could alleviate salinity stress in sweet pepper. To our knowledge, after well literature exploration, this work considers the first study examining the capability of green SNPs to alleviate salinity stress in sweet pepper plant. Indeed, this study seeks to keep sustainable agricultural practices that can alleviate the adverse effects of soil salinity on plant growth and ensure global food security.

## 2 Materials and method

### 2.1 SNPs synthesis using *Moringa oleifera* (Lam.) leaves extract

**Figure 1:**
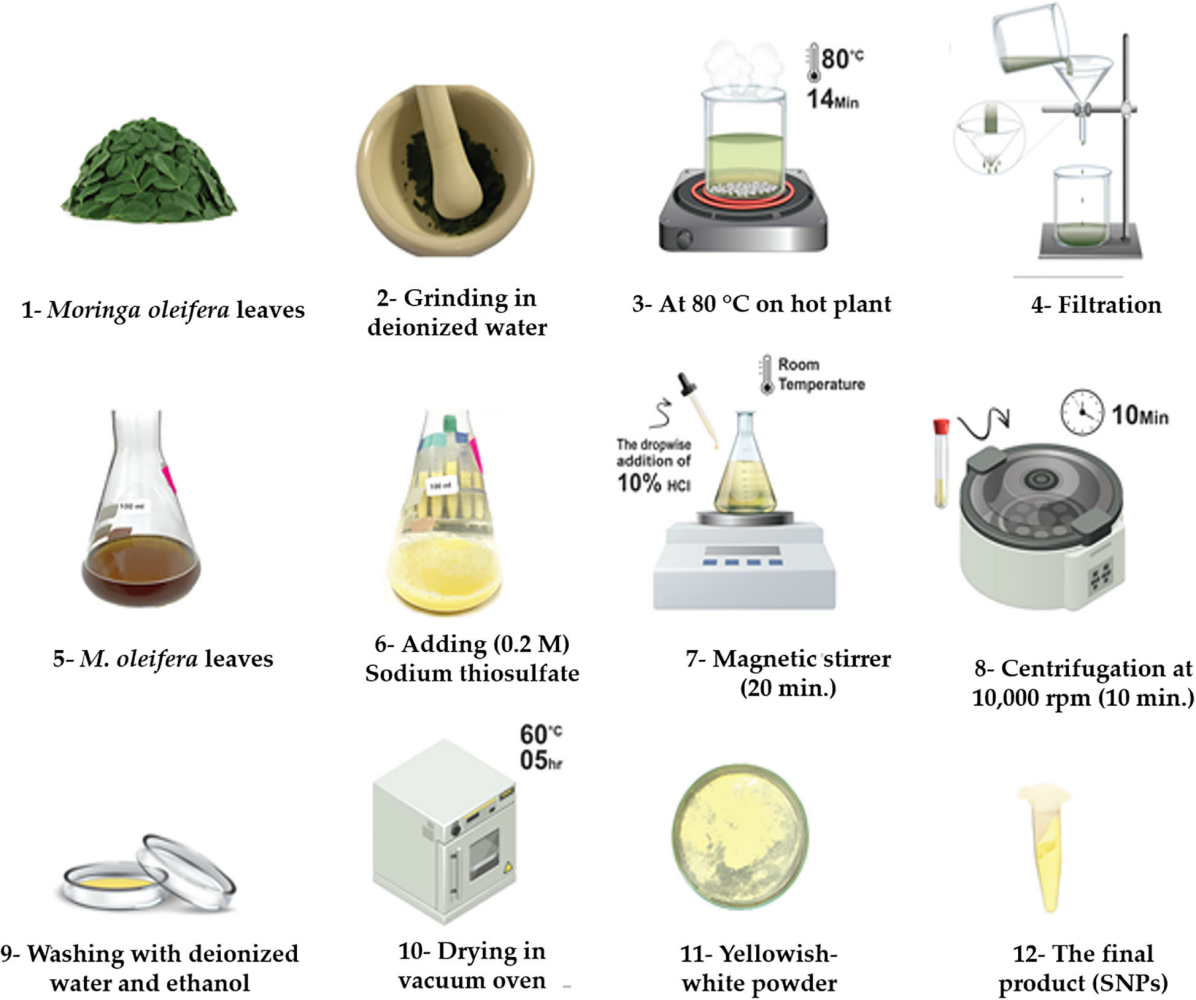
Flow chart for synthesizing SNPs using *Moringa oleifera* leaves extract.

The fresh leaves of *M. oleifera* were washed with running tap water to remove the impurities adhering to the surface. Then the leaves were gently wiped with filter paper, 50 g of leaves were added to 100 ml distilled water and grind, then kept at 80 °C on the hot plate for 14 min until the color of the water turned green. Then, the extract was cooled at room temperature, filtered, and stored in the refrigerator for SNPs synthesis. To synthesize SNPs, sodium thiosulfate pentahydrate (Na_2_S_2_O_3_.5H_2_O) was used. 5 g of sodium thiosulfate (0.2 M) solution was prepared by dissolving it in 100 ml of *M. oleifera* leaf extract and stirring gently for 20 min at room temperature. After adding 10% HCl dropwise while stirring and centrifuging at 10,000 rpm for 10 min, sulfur particles were precipitated. The precipitate was repeatedly cleaned with deionized water five times before being treated with absolute ethanol. The supernatant was disposed of. After gathering the product and drying it for five hours at 60 °C in a vacuum oven, a yellowish-white powder was produced that was then used for characterization (Ragab and Saad-Allah, 2020). The flowchart in **Figure 1** illustrates the process used to prepare SNPs. The synthesized SNPs were characterized by Fourier transform infrared (FTIR) [NICOLET iN10], X-ray diffraction (XRD), and transmission electron microscopy (TEM) [Mic JEM 1011] before usage in the sweet pepper treatment.

### 2.2 Experimental design and SNPs treatments

This experiment was carried out at King Abdulaziz University’s experimental station, Jeddah, Saudi Arabia (21°29’09.1’’N 39°14’31.0’’E), during the autumn of 2023, from October 9 to November 26. There was a slight wind and moderate humidity as the temperature gradually decreased. Overnight and early in the morning, the lowest temperature recorded within the greenhouse was 25**°**C, while the highest recorded temperature per day was 32**°**C. Three deionized water rinses were performed on sweet pepper seeds and then soaked in SNPs suspension at concentrations (1, 10, and 100 mg/l) in a petri dish for four hours in an incubator at 27**°**C. Then, germinated in 7 Kg size pot containers (with a 28 cm diameter and 22 cm height) filled with 5 kg of bitoms soil. The plants in the greenhouse were grown under natural light and consistently irrigated every two days with tap water at field capacity. After germination, the pots were thinned to contain five seedlings each. After the 14 days, third true was leaf appeared, then two levels of salinity stress were applied: (50 and 100 mM NaCl). Plant irrigated with tap water used as a control group. In this experiment, 3 x 4 factorial design was applied representing NaCl concentrations and SNPs treatment respectively. The details of the total 12 treatments shown in **Table 1**. Then, 47-day-old sweet pepper seedlings were harvested for plant growth traits determination. Plant leaves were stored at -80**°**C for physiological, biochemical and genetic analysis. Three duplicates of the experiment were conducted using a completely random design (CRD).

**Table 1:** The experimental design of the study.

No.	NaCl level (mM)	Sulfur nanoparticles treatment (mg/l)
1	0	0
2	50	0
3	100	0
4	0	1
5	50	1
6	100	1
7	0	10
8	50	10
9	100	10
10	0	100
11	50	100
12	100	100

### 2.3 Data collection

#### 2.3.1 Morphological traits

At the end of the experiment, shoot lengths were recorded for each plant. Shoots were weighed for fresh weight (FW), whereas dry weight (DW) was determined after drying plant samples in the oven at 65°C for 48 h (until constant weight is reached). In addition, shoot length (cm) was recorded. All measurements were taken from a sample of three plants from each treatment in each experiment.

#### 2.3.2 Physiological traits

The leaves’ total chlorophyll content was measured using the method outlined by Su et al. (2010). Chlorophyll was extracted by immersing 0.5 g of fresh leaf samples in 80% acetone in a shaker until the leaves lost color. A spectrophotometer was used to measure the total chlorophyll (both a and b) levels in the extract at 663 and 645 nm absorbance (A) by measuring the liquid above the sediment after the extract was centrifuged for 10 min at 13,000 rpm. The total chlorophyll content in the extract (mg/l) was calculated according to the following equation:


Chlorophyll content=20.31∗A663+8.05∗A645

Cell membrane stability, as indicated by electrolytes leakage (%), was measured using the method outlined by Alghabari et al. (2021). For this experiment, 100 mg of leaf bits were used in two tubes with 20 mL of deionized water each. One tube was incubated at 40°C for 30 min to test conductivity (C1), while the second tube was kept at 100°C for 10 min to measure conductivity (C2). The following formula was finally used to determine the electrolytes leakage (C):


$$\text{C}\left(\%\right)=1-\left(\frac{C1}{C2}\right)*100$$

Following Shah et al. (2022) methodology, the relative water content (RWC) of the leaves was calculated using the following formula:


$$\text{RWC}=\left[{\text{W}}_{Fresh}-{\text{W}}_{Dry}\right)/\left({\text{W}}_{Total}-{\text{W}}_{Dry}\right)\times 100]$$

Plant-water relations were evaluated using Sattar et al. (2020) methodology, including water potential (ψ_w_), osmotic potential (ψ_s_), and pressure potential (ψ_p_). Using a Scholander bomb (PMS Instrument Company, Albany, USA), the water potential of leaves (ψ_w_) was determined in accordance with the guidelines. Using an osmometer (Advanced Instruments, Norwood, USA), the osmotic potential (ψ_s_) was determined according to the predetermined method. As the difference between ψw and ψ_s_, the leaf pressure potential (ψ_p_) was calculated.

#### 2.3.3 Biochemical traits

Proline content in sweet pepper leaves was measured with ninhydrin using UV–vis spectrophotometer (DeNovix, United States, Product No. DS 11FX) according to the same pattern (Ábrahám et al., 2010). Valadez-Bustos et al. (2016) described a similar spectrophotometric method for measuring glycine betaine (GB) based on its reaction with iodine. Using the methodology of Hniličková et al. (2019), the Na^+^/K^+^ ration of the leaves was examined. Malondialdehyde (MDA) content reacts with thiobarbituric acid (TBA) to produce a pink chromogen that can be detected spectrophotometrically, which is the procedure for evaluating lipid peroxidation using MDA content. A standard curve calculates the sample’s MDA content, which is then expressed as nmol/mg protein (Reilly and Aust, 1999). The content of the superoxide anion radical (O_2_^-1^) was assessed using the method outlined by Ajiboye et al. (2016). Furthermore, the method described by Velikova et al. (2000) was used to estimate the concentration of hydrogen peroxide (H_2_O_2_).

The method outlined by Djanaguiraman et al. (2020) was used to evaluate the activity of antioxidant enzymes, including catalase (CAT), peroxidase (POD), and superoxide dismutase (SOD). Two grams of homogeneous frozen leaf samples were mixed with two milliliters of ice-cold 0.1 M Tris-HCl buffer for the study. Centrifugation was then performed for 15 min at 2,000°C and four °C. The supernatant was then collected, and enzymatic activity was measured according to manufacturer instructions using a SOD1 ELISA kit (Product No. MBS283325, MyBioSource, United States), POD using the peroxidase activity analytical kit (Product No. E-BC-K227-S, Elabscience, United States), and CAT assay kit (Product No. MBS8243260, MyBioSource, United States). Using their respective standard absorbance curves, the antioxidant enzymes’ activity was measured and expressed as units per milligram of protein (U/mg protein).

#### 2.3.4 Gene expression analysis

For gene-expression analysis the collected sweet pepper leaves (3^rd^ leaf) were immediately frozen in liquid-nitrogen and stored at -80 ^0^C till the start of the RNA extraction. RNeasy kit (Qiagen, Germany) used for RNA extraction according to the instruction provided. Total RNA of 2 µg was used to create cDNA library. QuantiTect reverse transcription kit (Qiagen, Germany) was used for this purpose. To check the transcript level of related genes in leaf tissues, the qRT-PCR was performed by a SYBR Green Kit (BioFACT, Korea), and the gene expression was normalized using TaActin1-expressing gene. For all qRT-PCR analysis, each expression profile was analysed and confirmed using three technical and three biological replicates as used by Li et al. (2018). Moreover, to calculate the relative gene expression for each sample double delta Ct value was used (Li et al., 2018). The list of gene primers used for qRT-PCR is given in **Table 2**. The images of the electrophoresis gel demonstrating the quality of the extracted RNA (**Supplementary Figure S1**), along with the amplification and melting curves (**Supplementary Figures S2**, **S3**) of the genes analyzed in real-time PCR are provided as **Supplementary Data**.

**Table 2:** The primers used in the estimation of relative gene expressions.

Primer	Forward	Reverse
*CaHAK6*	ACTTTGGAGGTTGGAATGATTTAC	AAGCTTTTTGCATTTGAAATACAA
*CaHAK7*	GGCTTCTAAGGGTTGGCATG	GCTTGCACACTTTATTTAAGGATT
*CaBiP1*	AGAGATCCCTCAGTAGCCAGC	GTTGTTCAACTCCTCAAAACGT
*CaBiP2*	AAGAAGTTGAGGCAGTGTGC	TGTGAATCGTCATCATCGTTG
*CaDHN3*	GAGTACCTGTACCAGCAGGA	TAATCTTCTCCTTTCTCCCC
*CaSOD*	TATGGAGCCTTAGAACCTGC	CCATTGAACTTGATAGCACCT
*CaPOD*	TCCTCCTCCTACTTCTAACC	ACAGACCTCTTTTGCTCACT
*CaCAT1*	TGTTGCTGGTGTTGGTGTTGGT	GCCTCTCCTAGACGGCCTTTCA
*CaPRP1*	AAGAGCCTCAGAGCGTGC	CCATGGACCTGGATTGCACCG
*CaActin*	AGGGATGGGTCAAAAGGATGC	GAGACAACACCGCCTGAATAGC

### 2.4 Statistical analysis

Statistics 8.1 software was utilized to analyze all data. Two-way analysis of variance (ANOVA) was performed to examine the effects of the studied salinity levels, SNPs treatments, and their interactions upon all investigated traits. Significant differences between treatments (p<0.05) were confirmed using Bonferroni multiple comparison test. All values were expressed with their standard error (SE) as a mean value of three replicates. RStudio version 1.3.959 was also used for the principal component analysis (PCA), correlation, and heatmap analyses.

## 3 Results

### 3.1 Characterization of the green synthesized SNPs

**Figure 2:**
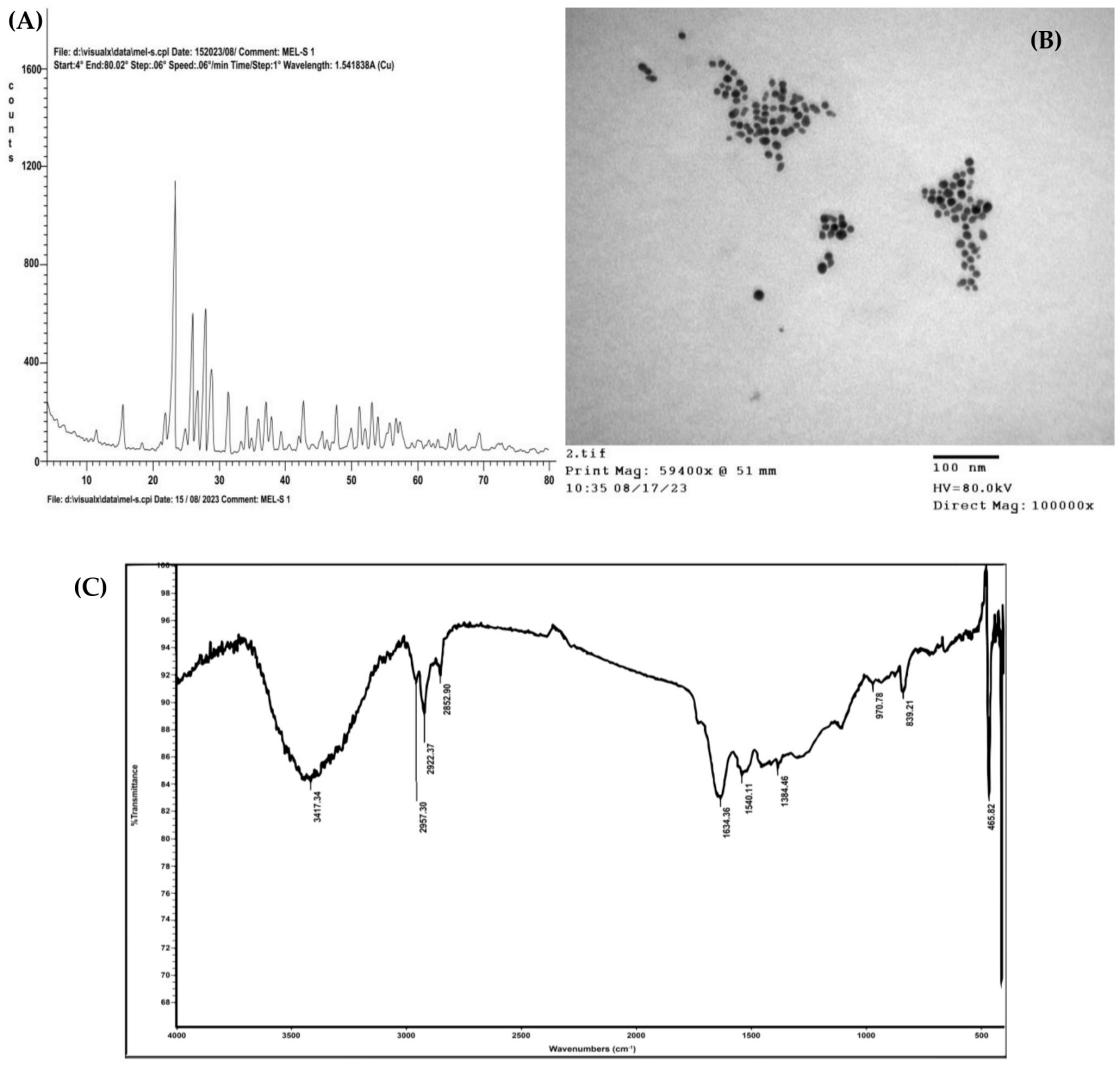
Characterization of greenly synthesized SNPs: **(A)** XRD pattern, **(B)** TEM micrograph, **(C)** FT-IR profile of *M. oleifera* leaves extract.

A yellowish-white powder was the result of greenly synthesizing SNPs using leaf extract from *Moringa oleifera*. The SNPs were successively synthesized, and their polycrystalline character was confirmed by the several sharp peaks visible in the XRD diffraction pattern (**Figure 2A**). The crystal planes of sulfur at 225, 193, 145, 599, 281, 617, 369, 274, 215, and 232 are responsible for the characteristic peaks at 2θ values of 15.33, 21.75, 22.94, 25.73, 26.56, 27.60, 28.64, 31.25, 34.07, and 36.97°, respectively. The significant locations and intensities of the diffraction peaks are in quite well match with standard sulfur (International Centre for Diffraction Data (ICDD), No. 00-008-0247). Using a transmission electron microscope, the formation of the morphological structure and SNPs was also observed (TEM). SNPs had a spherical shape, with an average size of 15 nm, according to TEM micrographs (**Figure 2B**). An FT-IR analysis was performed to identify the potential biomolecules in *M. oleifera* leaf extract that are responsible for stabilizing and capping the synthesized produced SNPs. *M. oleifera* leaf extract’s obtained FT-IR chart (**Figure 2C**) demonstrated a range of absorption peaks at 3417, 2957,2922, 2852, 1634, 1540, 1384, 970, 839, 465 and 411 cm^−1^.

### 3.2 Effect of SNPs application on sweet pepper morphological traits

**Figure 3:**
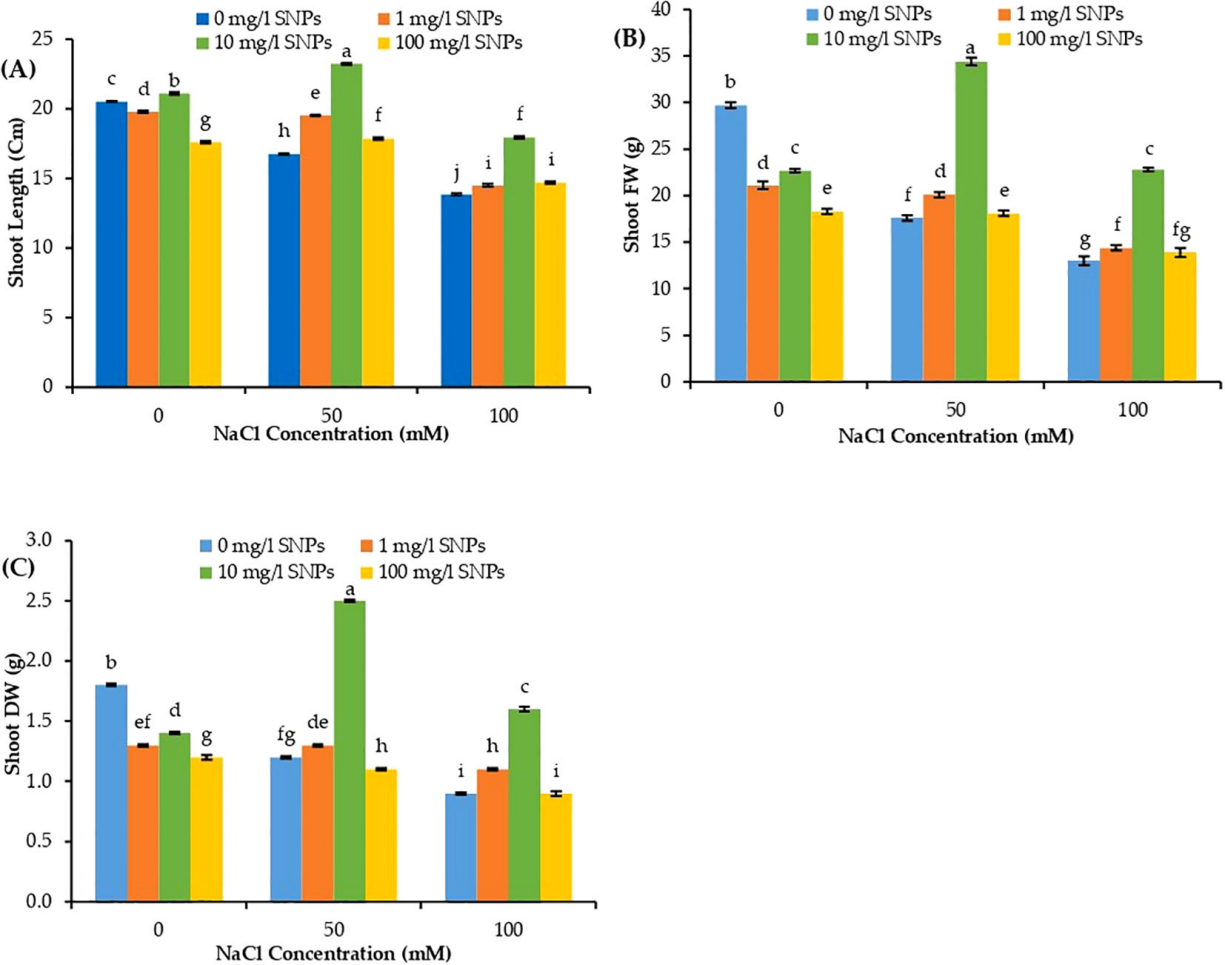
The interactive effect of seeds pre-soaking with different SNPs treatments, at different NaCl concentrations on morphological traits of sweet pepper: **(A)** length, **(B)** fresh weight (FW), **(C)** dry weight (DW). Graphs indicate the mean data analysed at p ≤ 0.05 during two factorial tri-replicate experiment. Different lowercase letters indicate significant differences within parameters (p < 0.05) as determined by Bonferroni multiple comparison test.

Shoot length increased significantly under all salt concentrations after treatment with 10 mg/l SNPs, as seen in **Figure 3A** when compared to the untreated control group. At both salt levels (50 and 100 mM NaCl), 1 mg/l SNPs treatment increased shoot length by about 16.52 and 4.61%, respectively, compared to untreated controls. Furthermore, at all salt concentrations (50 and 100 mM NaCl), 10 mg/l SNPs enhanced shoot length by around 38.6 and 29.6%, respectively. Conversely, at both salt levels (50 and 100 mM NaCl), applying 100 mg/l SNPs increased shoot length by roughly 6.56 and 6.06%, respectively (**Figure 3A**). As salt stress levels increased, sweet peppers’ shoot biomass decreased gradually, reaching its lowest value at 100 mM NaCl. **Figure 3B** shows the variation in the growth response of sweet pepper plants for shoot fresh weight (FW). All SNPs treatments reduced shoot FW under (0 mM NaCl) condition, as seen in **Figure 3B**. Under low salinity level (50 mM NaCl), shoot FW increased significantly when treated with 1 mg/l SNPs by about 14.20% higher than control. The treatment with 10 mg/l SNPs showed the most pronounced induction, increasing shoot FW relative to the corresponding controls by about 95.5% and 75.4% at salinity levels of 50 and 100 mM NaCl, respectively (**Figure 3B**).

According to **Figure 3C**, sweet pepper shoot dry weight (DW) significantly decreased by increasing salinity level reaching its minimum value (50% lower than non-salinized control) at 100 mM NaCl. Compared to the untreated stressed controls, all investigated SNPs treatments significantly increased shoot DW at all salinity levels. One mg/l SNPs treatment increased shoot DW by 8.3% and 22.22%, higher than their corresponding controls under salinity levels 50 and 100 mM NaCl respectively. SNPs (10 mg/l) enhanced shoot DW by about 108.3% and 77.8%, respectively, at both salt levels (50 and 100 mM NaCl), Higher than their corresponding controls (**Figure 3C**).

### 3.3 Effect of SNPs application on sweet pepper physiological traits

#### 3.3.1 Chlorophyll and cell membrane stability

**Figure 4:**
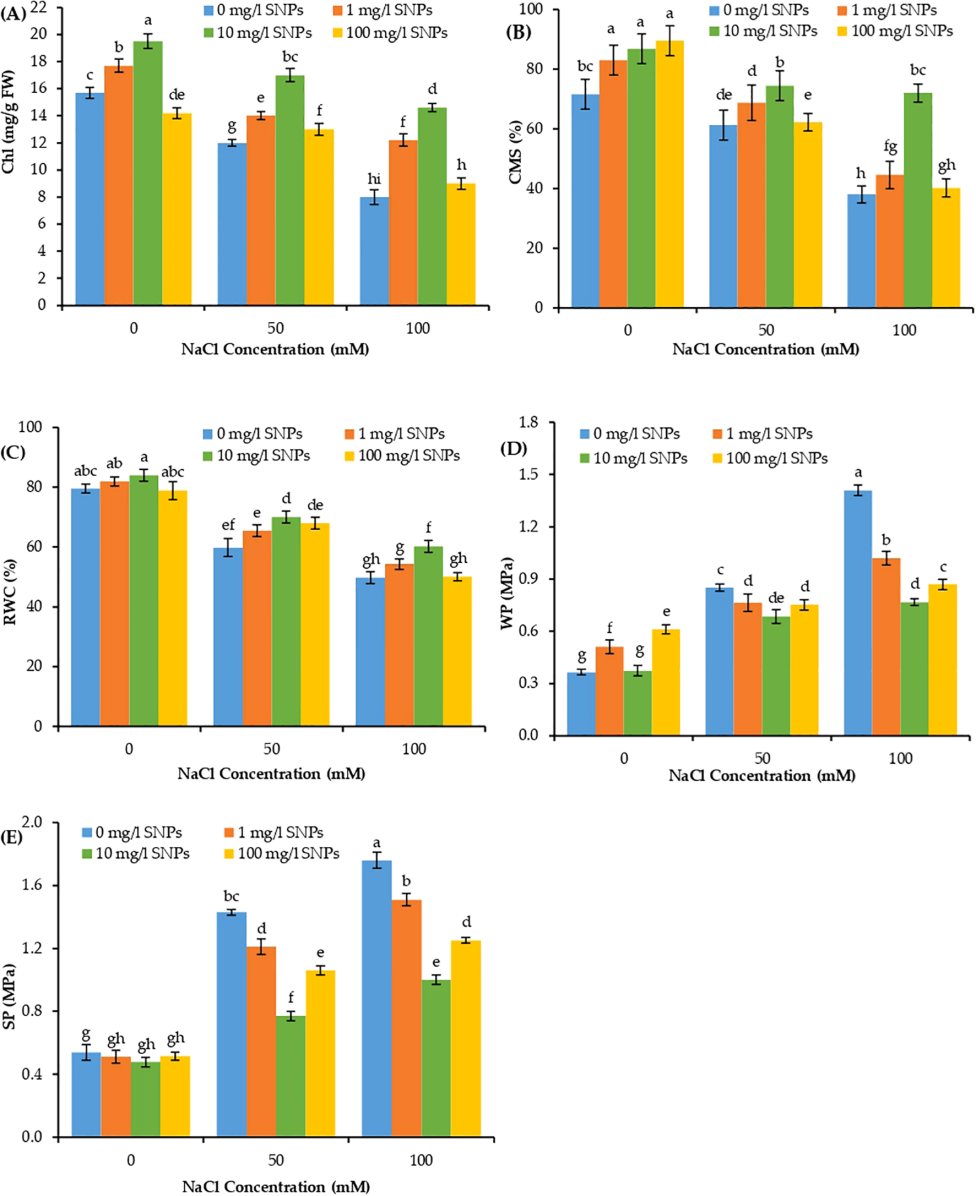
The interactive effect of seeds pre-soaking with different SNPs treatments, at different NaCl concentrations on physiological traits of sweet pepper: **(A)** Chlorophyll content (Chl), **(B)** Cell membrane stability (CMS), **(C)** relative water content (RWC), **(D)** water potential (WP), **(E)** solute potential (SP). Graphs indicate the mean data analysed at p ≤ 0.05 during two factorial tri-replicate experiment. Different lowercase letters indicate significant differences within parameters (p < 0.05) as determined by Bonferroni multiple comparison test.

Both total chlorophyll (Chl) content and cell membrane stability (CMS) revealed significant variation due to individual and interaction effects of salinity and SNPs treatments. Increasing salinity levels significantly reduced Chl and CMS percentages in sweet pepper leaves, with the maximum at 100 mM NaCl and minimum at 0 mM NaCl (**Figures 4A, B**). Under 100 mM NaCl condition, Chl content and CMS percentage reduced by about 49 and 46.9% lower than their corresponding values in non-saline control. On the other hand, Chl and CMS illustrated a significant increase by increasing the levels of SNPs treatments from 1 to 10 mg/l. Moreover, for the interaction effect of salinity and SNPs, the treatment with 10 mg/l SNPs demonstrated a significant increase in Chl and CMS at all salinity levels in sweet pepper. Seed presoaking with 10 mg/l SNPs enhanced Chl content and CMS percentage by about 82.5 and 89.5% respectively higher than untreated control under the highest saline condition (100 mM NaCl).

#### 3.3.2 Plant water relations

The water-related parameters such as relative water content (RWC), water potential (WP), and solute potential (SP) demonstrated significant variations due to individual and interaction effects of both factors of treatments including salinity and SNPs treatments (**Figures 4C–E**). Shoot RWC decreased significantly with increasing salt concentration, reaching values about 24.7 and 37.4% lower than unstressed control when treated with 50 and 100 mM NaCl respectively (**Figure 4C**). Conversely, the WP and SP depicted a significant rise with increasing salinity levels, reaching values about 3 and 4 folds that of control respectively at 100 mM NaCl (**Figures 4D, E**). On the other hand, the RWC manifested a significant increase with increasing levels of SNPs from 1 to 10 mg/l. Besides, at the same concentrations of SNPs, the WP and SP showed the converse trend as compared to RWC with a consistent decline from 1 to 10 mg/l. Moreover, for the combined effect of salinity and SNPs (salinity x SNPs), 10 mg/l SNPs depicted a significant increase in RWC at all levels of salt stress and a significant decrease in WP and SP at all levels of salt stress. Under 100 mM NaCl, seed priming with 10 mg/l SNPs increased RWC by about 20.9% more than control, while reduced WP and SP by about 45.5 and 43.2% lower than untreated control respectively (**Figures 4C–E**).

### 3.4 Effect of SNPs application on sweet pepper biochemical traits

#### 3.4.1 Osmolytes

**Figure 5:**
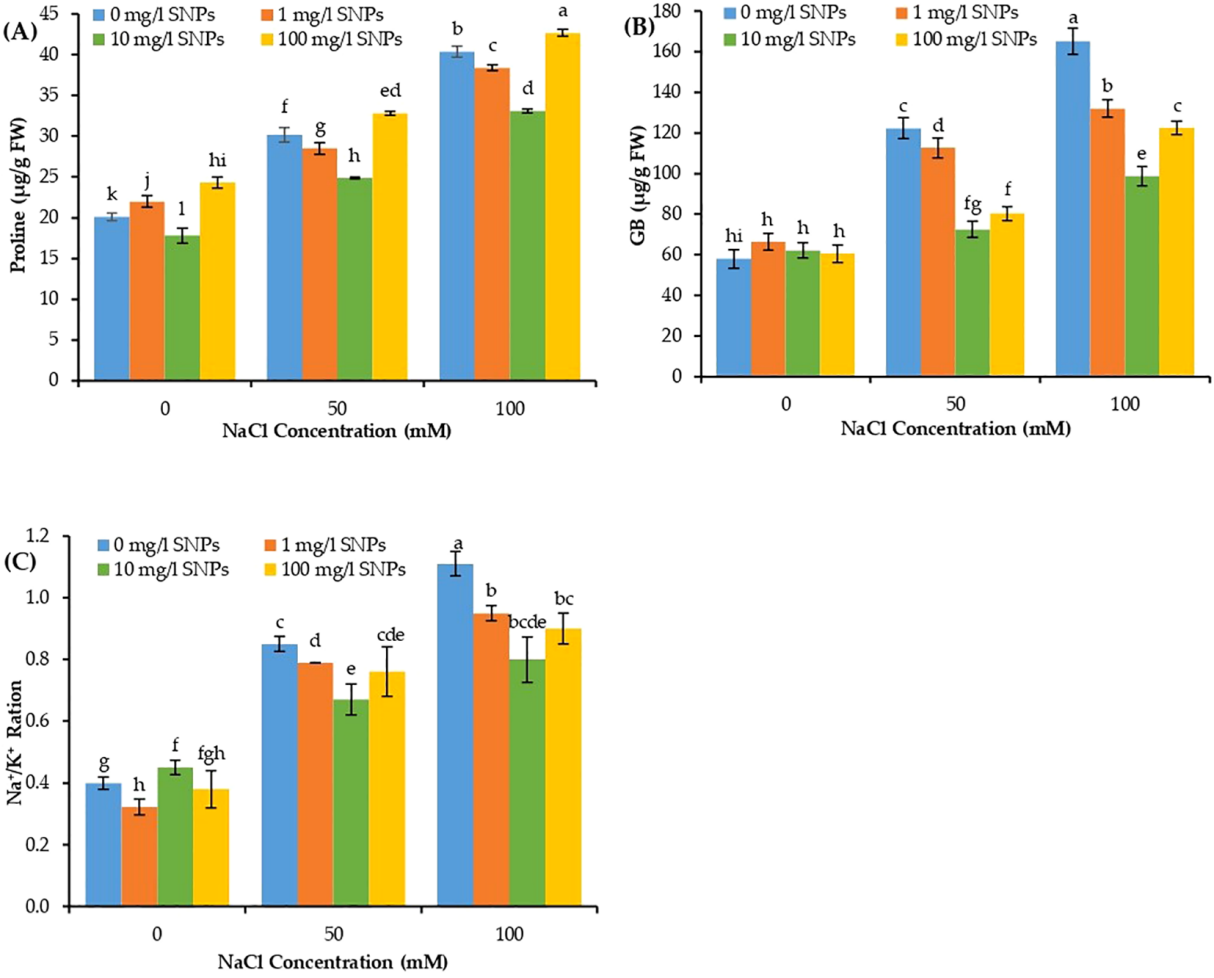
The interactive effect of seeds pre-soaking with different SNPs treatments, at different NaCl concentrations on biochemical traits of sweet pepper: **(A)** proline, **(B)** glycine betaine (GB), **(C)** Na^+^/K^+^ ratio. Graphs indicate the mean data analysed at p ≤ 0.05 during two factorial tri-replicate experiment. Different lowercase letters indicate significant differences within parameters (p < 0.05) as determined by Bonferroni multiple comparison test.

The osmolytes such as proline and GB, in addition to Na^+^/K^+^ ratio varied significantly due to individual and interaction effects of salinity and SNPs treatments. The proline, GB, and Na^+^/K^+^ ratio showed a significant increase in concentration with increasing levels of salt stress, with the maximum at 100 mM NaCl and minimum at control (0 mM NaCl) (**Figure 5**). Proline and GB concentration and Na^+^/K^+^ ratio increased dramatically by about 101, 185.5 and 177.5% respectively higher than non-saline control when treated with 100 mM NaCl. On the other hand, the aforementioned traits manifested a significant decline due to seed priming with SNPs under both investigated salinity levels. Moreover, for the interaction effect of salinity and SNPs (salinity x SNPs), the SNPs treatment 10 mg/l depicted the most pronounced significant decrease in proline, GB and Na^+^/K^+^ ratio, at both concentrations of salinity. Ten mg/l SNPs reduced proline, GB content and Na^+^/K^+^ ratio by about 18.1, 40.3 and 27.7% respectively lower than untreated control at 100 mM NaCl. The decline in Na^+^/K^+^ ratio at 100 mg/l level of SNPs was due to an increasing influx of K^+^ ion (**Figure 5C**).

#### 3.4.2 Oxidative stress biomarkers

**Figure 6:**
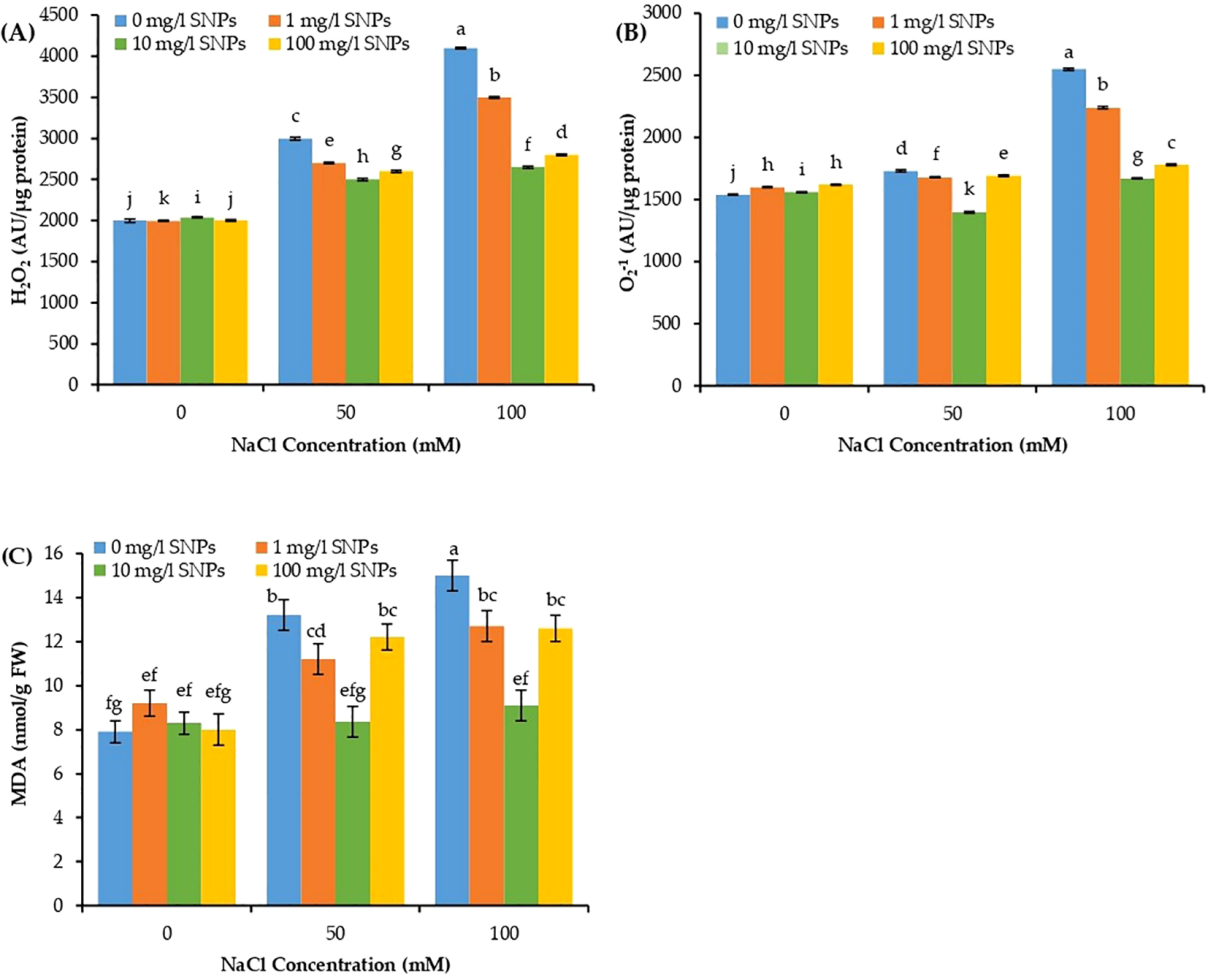
The interactive effect of seeds pre-soaking with different SNPs treatments, at different NaCl concentrations on free radicals’ concertation of sweet pepper: **(A)** Hydrogen peroxide (H_2_0_2_), **(B)** Superoxide anion (O_2_^-1^), **(C)** Malondialdehyde (MDA). Graphs indicate the mean data analysed at p ≤ 0.05 during two factorial tri-replicate experiment. Different lowercase letters indicate significant differences within parameters (p < 0.05) as determined by Bonferroni multiple comparison test.

The concentration of ROS, for instance, H_2_O_2_ and superoxide anion (O_2_^-1^), in addition to malondialdehyde (MDA) varied significantly due to individual and combined effects of SNPs and salinity treatments. Increasing the salinity level significantly increased the concentration of H_2_O_2_, O_2_^-1^ and MDA in sweet pepper leaves, recording maximum values (one-fold each) at 100 mM NaCl compared to unstressed control (**Figure 6**). However, these stress markers manifested a dramatic decrease with increasing levels of SNPs. Moreover, for the interaction effect of salinity and SNPs (salinity x SNPs), all SNPs treatments demonstrated a significant decline in H_2_O_2_, O_2_^-1^ and MDA at both salinity concentrations. It is worth to motion that, seed presoaking with 10 mg/l SNPs recorded the minimum values for H_2_O_2_, O_2_^-1^ and MDA in sweet pepper leaves by about 35.4, 34.5 and 39.3% lower than untreated control under the highest salinity condition (**Figures 6A–C**).

#### 3.4.3 Antioxidant enzymes

**Figure 7:**
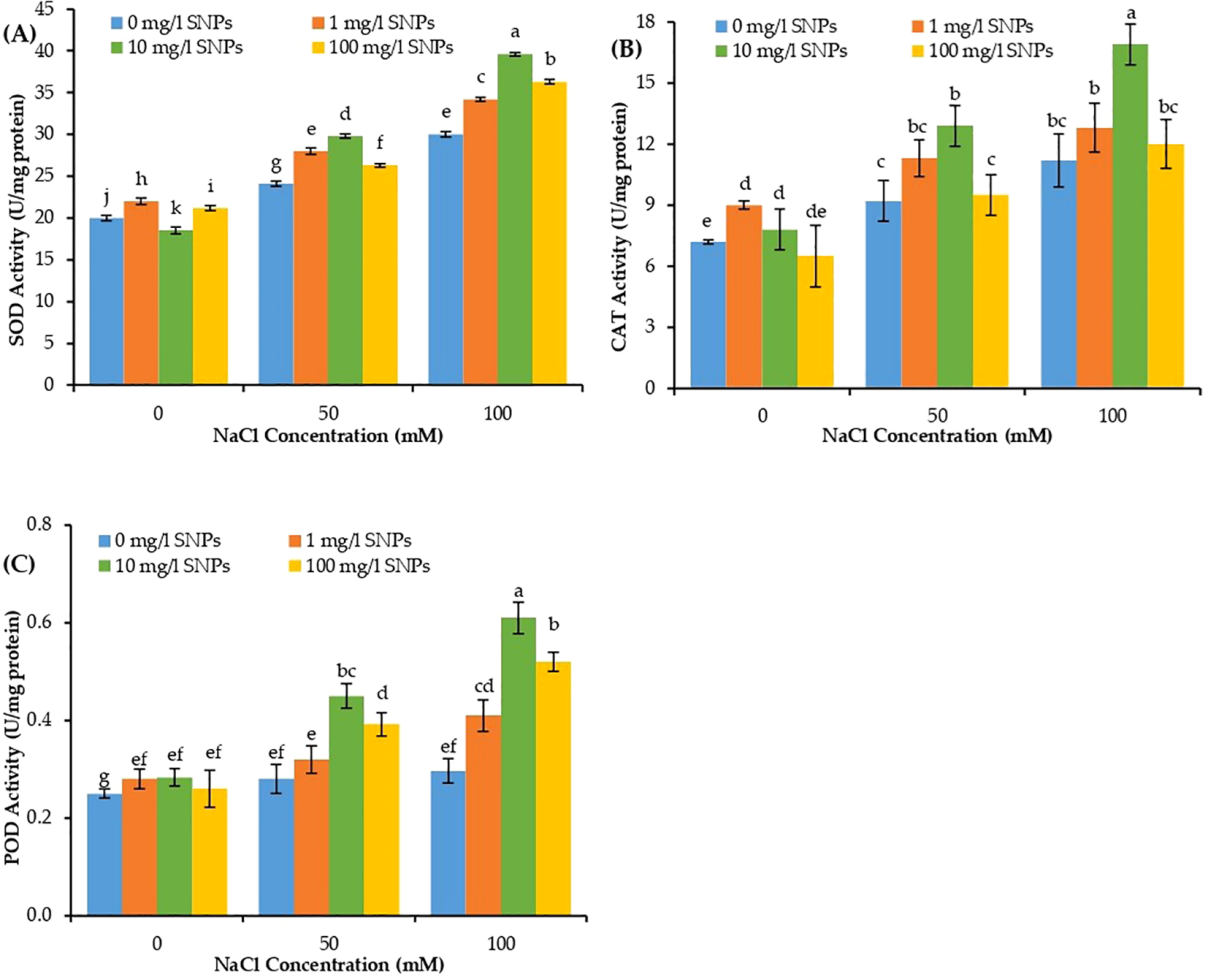
The interactive effect of seeds pre-soaking with different SNPs treatments, at different NaCl concentrations on antioxidant enzymes activity of sweet pepper: **(A)** superoxide dismutase (SOD), **(B)** catalase (CAT), **(C)** peroxidase (POD). Graphs indicate the mean data analysed at p ≤ 0.05 during two factorial tri-replicate experiment. Different lowercase letters indicate significant differences within parameters (p < 0.05) as determined by Bonferroni multiple comparison test.

The catalytic activities of antioxidant enzymes SOD, POD, and CAT varied significantly in sweet pepper leaves due to individual and combined effects of salt and SNPs treatments. The activities of the three studied antioxidant enzymes increased significantly in response to salinity stress (**Figure 7**). At 100 mM NaCl, the activity of SOD, CAT and POD increased by about 50, 55.6 and 18.4% correspondingly higher than non-salinized control. Furthermore, the activities of antioxidant enzymes depicted a consistent increase in response to SNPs treatments mainly under saline conditions. The interaction effect of NaCl and SNPs illustrated significant variation in the catalytic activities of all investigated enzymes. SNPs treatment 10 mg/l demonstrated a significant increase in the catalytic kinetics of all antioxidant enzymes at both levels of NaCl in sweet pepper. The most pronounced effect for SNPs treatments recorded for 10 mg/l under 100 mM NaCl, where the activity of SOD, CAT and POD increased by about 32, 50.9 and 106.1% subsequently higher than untreated stressed plants (**Figures 7A–C**).

### 3.5 Effect of SNPs application on sweet pepper salt tolerant genes expression

**Figure 8:**
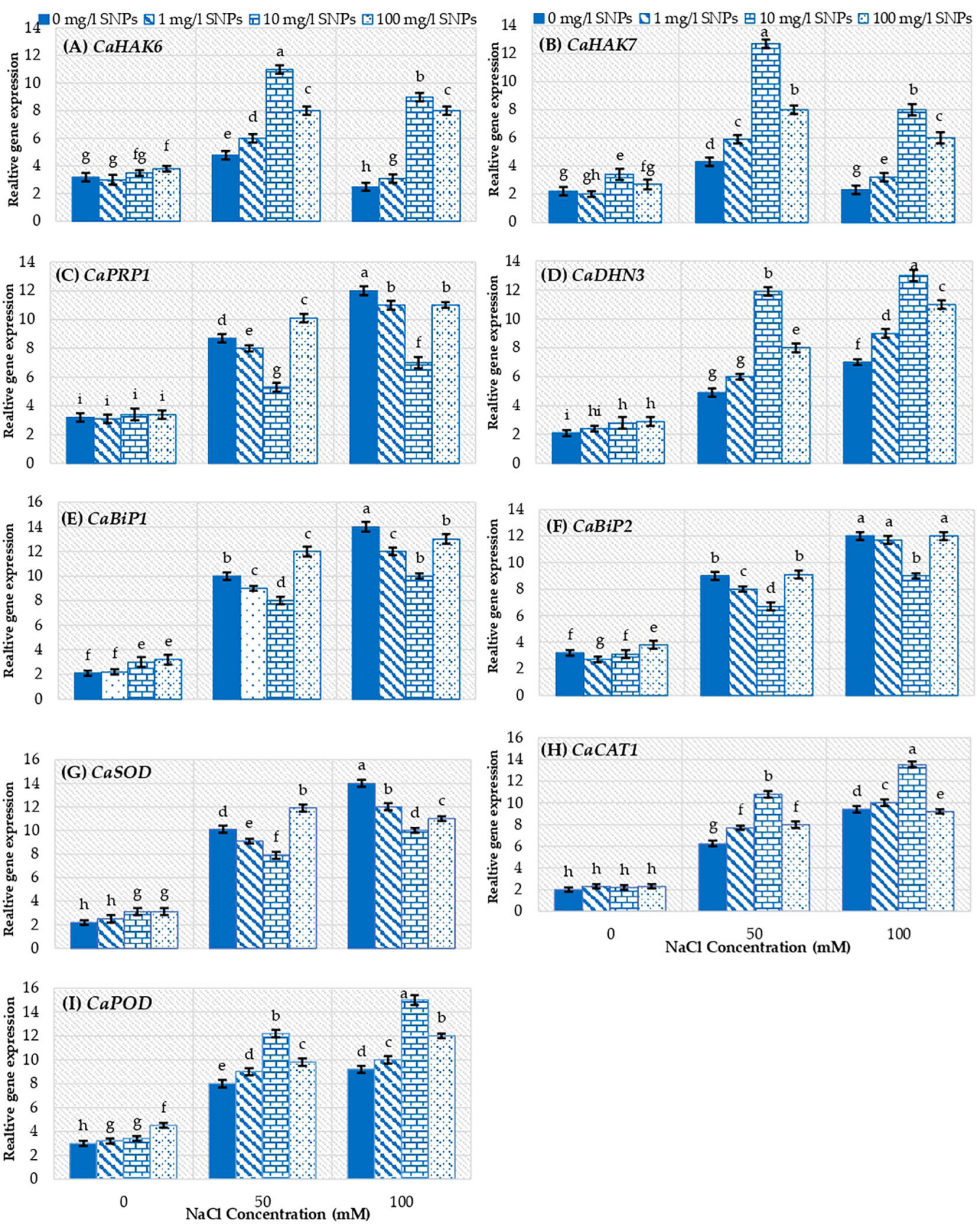
The interactive effect of seeds pre-soaking with different SNPs treatments, at different NaCl concentrations on the relative expression of some genes in sweet pepper. **(A)** *CaHAK6*, **(B)** *CaHAK7*, **(C)** *CaPRP1*, **(D)** *CaDHN3*, **(E)** *CaBiP1*, **(F)** *CaBiP2*, **(G)** *CaSOD*, **(H)** *CaCAT1*, **(I)** *CaPOD*. Different lowercase letters indicate significant differences within parameters (p < 0.05) as determined by Bonferroni multiple comparison test.

Gene expression analyses showed that, relative expression of genes *CaHAK6* and *CaHAK7*, regulating the influx of K^+^, depicted significantly (p ≤ 0.05) varying levels of transcripts under varying interactions of salinity and SNPs (**Figures 8A, B**). The relative expression of *CaHAK6* and *CaHAK7* upregulated significantly only when treated with 50 mM NaCl by about 50 and 95.5% respectively higher than unstressed control. The SNPs treatment 10 mg/l followed by 100 and 1 mg/l has significantly increased the expression of *CaHAK6* and *CaHAK7* under saline conditions. The most remarkable induction recorded for 10 mg/l SNPs at 50 mM NaCl, where the expression level of *CaHAK6* and *CaHAK7* increased by about 2 and 1.3 folds respectively higher than untreated plants. Relative expression of the *CaPRP1*, regulating the accumulation of proline, showed a significant (p ≤ 0.05) enhancement in relative expression in sweet pepper leaves under both saline conditions. However, SNPs treatments did not enhance *CaPRP1* expression level under both non-saline and saline conditions. (**Figure 8C**). Besides, the ROS scavenging gene *CaDHN3* expression level increased by about 1.3 and 2.3 folds in response to 50 and 100 mM NaCl treatments correspondingly compared to non-salinized control. *CaDHN3* recorded maximum relative expression at SNPs level 10 mg/l followed by100 and 1 mg/l at both salinity concentrations (**Figure 8D**). 10 mg/l SNPs enhanced *CaDHN3* expression level by about 142.9 and 85.7% higher than untreated stressed plants at 50 and 100 mM NaCl, respectively. On the other hand, *CaBiP1* and *CaBiP2* genes positively impart plant tolerance to salinity stress by reducing ROS accumulation, MDA content and increasing the relative water content. Interestingly these genes showed maximum relative expression in response to salinity treatments in sweet pepper leaves (**Figures 8E, F**). Moreover, treatment with 100 mg/l SNPs further increased *CaBiP1* relative expression by about 20% higher than untreated stressed control at 50 mM NaCl (**Figure 8E**).

The relative expression of *CaSOD* showed significant (p ≤ 0.05) difference under saline conditions (**Figure 8G**) as compared to non-salinized control. The level of *CaSOD* transcripts was 3.6 and 5.4 folds that in control at 50 and 100 mg/l NaCl respectively. Only 100 mg/l SNPs could enhance *CaSOD* expression level under 50 mM NaCl. Furthermore, the expression of *CaSOD* was consistent with the activity of SOD enzyme in response to salinity stress. On the other hand, *CaCAT1 and CaPOD* illustrated significantly (p ≤ 0.05) different expression under varying saline concentrations due to changing levels of SNPs (**Figures 8H, I**) as compared to control. At all salinity concentrations, the genes *CaCAT1 and CaPOD* recorded maximum expression at 10 mg/l SNPs followed by100 and 1 mg/l. Seed priming with 10 mg/l SNPs increased the relative expression of *CaCAT1 and CaPOD* by about 41.6 and 63% higher than untreated stressed control at 100 mM NaCl, subsequently (**Figures 8H, I**).

### 3.6 Statistical interpretations between physiological and biochemical traits

#### 3.6.1 Correlation

**Figure 9:**
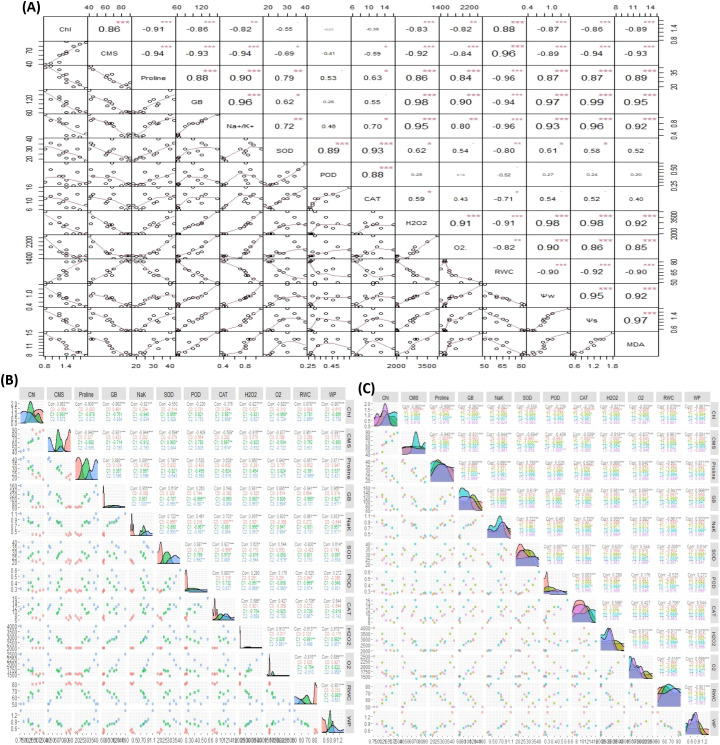
The correlogram illustrating the effect of: **(A)** Salinity and SNPs treatment, **(B)** Individual salinity concentrations, **(C)** Individual concentrations of SNPs on the paired association of antioxidant enzymes, Chl, CMS, ROS, osmolytes, and plant water relations in sweet pepper leaves. CAT, Catalase; SOD, Superoxide dismutase; POD, Peroxidase; MDA, malondialdehyde; GB, Glycine Betaine; CMS, Cell Membrane Stability; RWC, Relative water content; WP, Water potential; SP, Solute potential. ***Significant at p ≤ 0.001; **Significant at p ≤ 0.01; *Significant at p ≤ 0.05.

The overall correlation analysis of antioxidant enzymes, Chl, CMS, ROS, osmolytes, and plant water relations in sweet pepper leaves has illustrated a significant and varying extent of paired association among them. Overall, the significance of paired association among traits changed with both the type and level of treatments. The Chl showed a strong positive paired association with CMS and RWC and a negative paired association with GB, proline, Na^+^/K^+^, MDA, ROS, WP, and SP as indicated in **Figure 8A**. Consistent with Chl, the CMS varied in opposite directions with ROS, osmolytes content, and plant water relations including WP, and SP (**Figure 9A**). On the other hand, the SOD activity depicted a positive association with GB, proline, and Na+/K+ (**Figure 8A**). Furthermore, the catalytic activity of CAT varied in a positive direction with proline and Na^+^/K^+^ ratio (**Figure 9A**). In general, the ROS, activity of antioxidant enzymes (SOD, POD, CAT), MDA, osmolytes (GB and proline), Na^+^/K^+^, WP, and SP depicted significant positive association among them as shown in **Figure 9A**. However, the aforementioned parameters illustrated a negative correlation with Chl and CMS (**Figure 9A**).

Besides, the correlation analysis concerning the individual effect of salinity and SNPs treatments in sweet pepper leaves showed that both treatments affect the paired association of traits in different ways (**Figures 9B, C**). The correlation analysis for the individual effect of salinity treatments indicated that all treatments impact the paired association of traits in different ways which proved that the association of traits is highly vulnerable to the concentration of salinity (**Figure 9B**). Correspondingly, the correlogram analysis for the effects of SNPs treatment has proved that the paired association of traits is highly vulnerable to the concentration of treatments (**Figures 9B, C**).

#### 3.6.2 Principal component analysis

**Figure 10:**
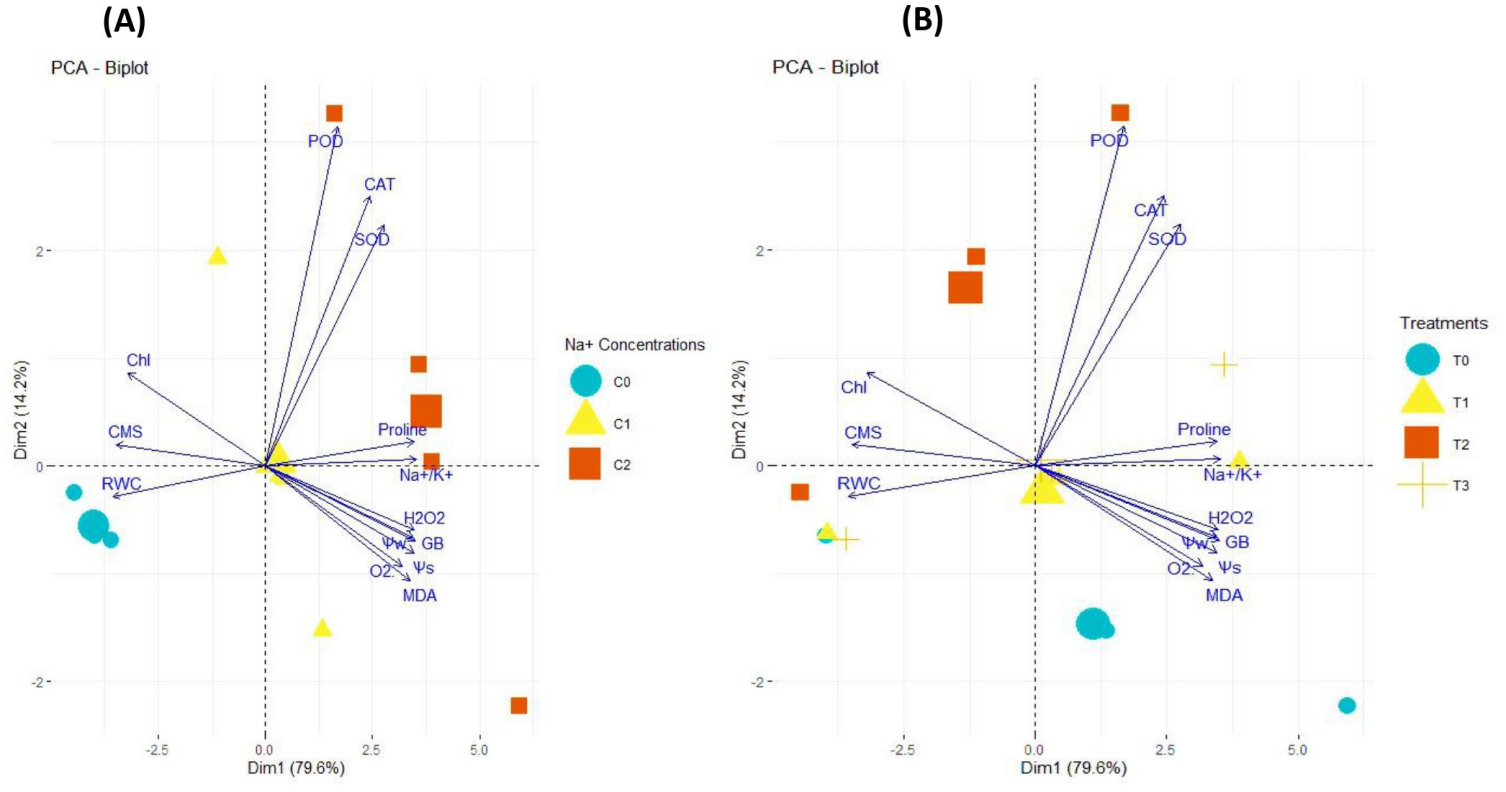
PCA biplot illustrating varying impacts of: **(A)** different salinity concentrations (C0 = 0 mM, C1 = 50 mM, C3 = 100 mM), **(B)** different SNPs levels (T0 = 0 mg/l, T1 = 1 mg/l, T3 = 10 mg/l, T4 = 100 mg/l) on the extent of association and expression of antioxidant enzymes, Chl, CMS, ROS, osmolytes, and plant water relations in sweet pepper leaves. These parameters were influenced by both individual SNPs and salinity treatments, as well as their combined effects.

The PCA biplot analysis illustrated the differential impacts of salinity concentrations and SNPs treatments on the expression and association of antioxidant enzymes, Chl, CMS, ROS, osmolytes, and plant water relations in sweet pepper leaves. The PCA biplot analysis for the salt treatments illustrated the varying impacts of different salinity concentrations (0, 50 and 100 mM NaCl) on the extent of association and expression of antioxidant enzymes, Chl, CMS, ROS, osmolytes, and plant water relations (**Figure 10A**). The varying lengths of vectors from biplot origin indicate that each trait is affected differently due to salinity. Furthermore, the distance between trait vectors indicates the extent of paired association among the traits. The closer vectors indicated that traits are more closely associated and vice versa. Besides, the varying distribution of salinity concentrations in biplot quadrants indicates that each concentration of salinity is affected differently by the magnitude and the type of trait association. Similarly, the biplot analysis for the effectiveness of SNPs treatments (0, 1, 10 and 100 mg/l) also proved that the response of traits varied with the varying levels of SNPs (**Figure 10B**). Corresponding to salinity, the varying lengths of vectors from biplot origin proved that each trait is differently affected by SNPs treatments. The varying distribution of SNPs concentrations in biplot quadrants proved that the variation in the degree of trait correlation is highly associated with the levels of SNPs. Furthermore, **Table 3** outlines the PCA components, their respective contributions and the attributes they encompass. PC1 (70.8%) primarily represents stress responses, strongly associated with Na^+^/K^+^ ratio, proline, GB, MDA, H_2_O_2_, WS, and antioxidant enzymes (CAT, POD, SOD). PC2 (14.2%) captures variations in chlorophyll content, CMS, and RWC, indicating differences in water retention and membrane stability. 10 and 100 mg/l SNPs exhibit stronger stress responses, while 0 and 1 mg/l SNPs show relatively lower impacts. The clear separation along PC1 and PC2 highlights the physiological adaptations under varying NaCl levels.

**Table 3:** PCA components, their respective contributions and the attributes.

Principal Component	Percentage Contribution	Key Attributes
PC1 (Dim1)	70.8%	Na^+^/K^+^, Proline, GB, H_2_O_2_, O_2_, WS, MDA, CAT, POD, SOD
PC2 (Dim2)	14.2%	Chl, CMS, and RWC
PC3 (Dim3)	Minor Contribution	Likely minor variations in metabolic responses

#### 3.6.3 Heatmap analysis

**Figure 11:**
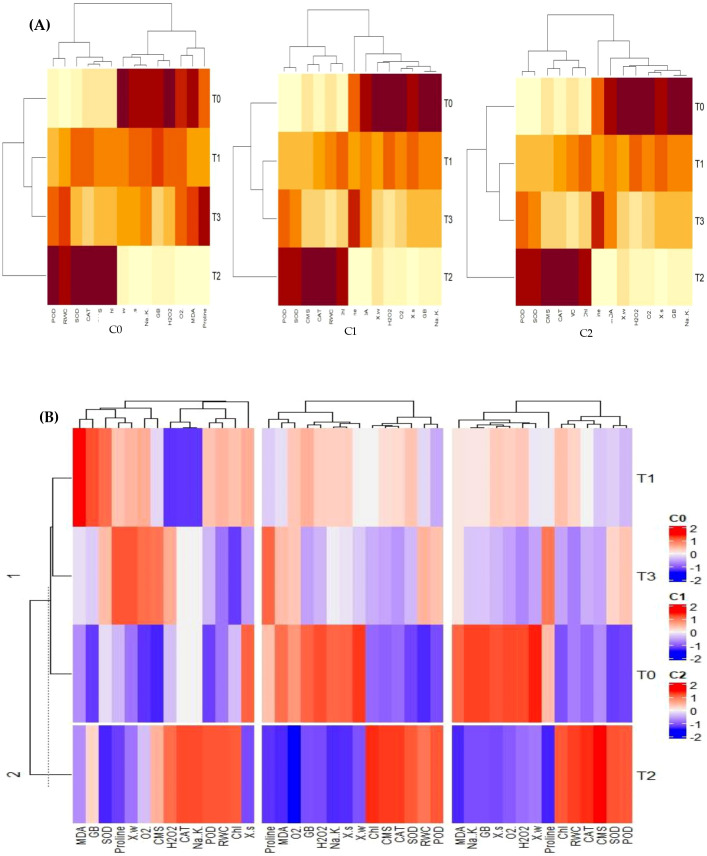
Different band patterns and cluster distribution of: **(A)** individual heatmaps, **(B)** combined heatmap that expression and association of antioxidant enzymes, Chl, CMS, ROS, osmolytes, and plant water relations in sweet pepper leaves are different for the same levels (T0 = 0 mg/l, T1 = 1 mg/l, T3 = 10 mg/l, T4 = 100 mg/l) of SNPs under varying concentrations of salinity stress (C0 = 0 mM, C1 = 50 mM, C3 = 100 mM). CAT, Catalase; SOD, Superoxide dismutase; POD, Peroxidase; MDA, malondialdehyde; GB, Glycine Betaine; CMS, Cell Membrane Stability; RWC, Relative water content; WP, Water potential; SP, solute potential).

Both individual and combined heatmap cluster analyses have further endorsed the results of PCA. The different band patterns and clusters distribution of heatmap indicated that expression and association of antioxidant enzymes, Chl, CMS, ROS, osmolytes and plant water relations in sweet pepper leaves are different for the same levels (T0 = 0 mg/l, T1 = 1 mg/l, T3 = 10 mg/l, T4 = 100 mg/l) of SNPs under varying concentrations of salinity stress (C_0_ = 0mM, C_1_ = 50 mM, C_3_ = 100 mM) as shown in **Figure 11A**. This has proved that varying interactions of salinity and SNPs (salinity x SNPs) influence the correlation and expression of traits in different ways. In fact, this was the further rectification of the results from PCA. These results were also endorsed by the combined heatmap analysis that explicated that each varying interaction of salinity and SNPs (salinity x SNPs) impacts the expression and association of traits in different ways (**Figure 11B**).

## 4 Discussion

Sulfur is an essential macronutrient for plants, and it has many important functions related to plant metabolism including coenzyme activity, production of metabolites, and essential amino acids (Hasanuzzaman et al., 2018). Therefore, it may be accountable for numerous facets of a plant’s defense mechanism against environmental stress (Capaldi et al., 2015). Many agricultural systems use essential elements that contain NPs because of their special physicochemical qualities, which include a high surface area to volume ratio, high catalytic potential, and capacity to engineer electron exchange (Najafi et al., 2020). The synthesis of plant-based nano-fertilizers is an excellent approach due to their low cost, non-pathogenic factor, non-toxic phytochemical constituents, flexibility in reaction parameters, and biochemical diversity of plant extract (Mahakham et al., 2016). Nano-fertilizers slowly release nutrients to the plants, thereby preventing excessive nutrient loss. NPs are biologically inert and non-destructive, allowing them to persist in soil for extended periods. This can lead to alterations in soil microflora populations, soil fertility, and the metabolism and physiology of plant species. The interactions demonstrate noteworthy impacts on the accessibility, transportation, and mobility of NPs in the soil system (Rehman et al., 2024). Compared to traditional fertilizers, they can be more effective mainly when they are green synthesized to scure the agricultural sustinabilty. Nanoparticles enhance crop tolerance to salt by modifying hormone levels, oxidative enzyme activities, ion homeostasis, gene expression, and defense mechanisms (Zulfiqar and Ashraf, 2021; Fouda et al., 2024). The implications may vary according to the environmental conditions or types of plants, in addition to the shape, quantity, and size of NPs applied (Wahid et al., 2020).

In the current study, *Moringa oleifera* leaves’ extract was used to create a new approach for SNPs biosynthesis. The characteristic diffraction peaks in the XRD pattern indicated the polycrystalline structure of the green biosynthesized SNPs, which aligned with standard sulfur. The average crystallite size of the formed SNPs was determined to be 15 nm (Ragab and Saad-Allah, 2020). The undesignated peaks in the XRD pattern represent the crystalline and amorphous organic biomolecules present in the extract of *M. oleifera*. The bio-synthesized SNPs’ XRD analyses were similar to the previous study reported by Kouzegaran and Farhadi (2017). Additionally, TEM micrographs demonstrated that the produced SNPs had a spherical morphology and perfectly matched the results of the XRD investigation. According to the FTIR spectrum of SNPs, the absorption peaks at 2922 cm^−1^ may be caused by the alkanes’ C–H stretching, while the intense peak at 3417 cm^−1^ may be the result of alcohols’ O–H groups and amines’ N–H groups (Akintelu and Folorunso, 2019). The FTIR spectrum showed a band at 1634 cm^−1^, caused by the amide proteins’ carbonyl groups (C=O) vibrating (Salam et al., 2014). Thus, FTIR analysis of the synthesized SNPs confirmed the existence of unique peaks corresponding to vibrational bands of hydroxyl (^-^OH), amine (N-H), and carbonyl groups (C=O) as functional groups. Based on the above observations, it can be inferred that in addition to proteins in *M. oleifera*, aromatic molecules such as phenolic and flavonoids facilitate the reduction of S ions into SNPs and their stability. Comparable results were reported by Malapermal et al. (2017) for silver NPs and by Tripathi et al. (2018) for SNPs.

Salinity application in the pot experiment led to a remarkable decrease in sweet pepper growth, as shown by a reduction in shoot length, fresh weight and dry weight. The growth inhibition evident in plants is directly caused by salinity stress and accumulation of Na and Cl ions in their tissues and the disruption of essential metabolic activities such as photosynthesis and the uptake of nutrients (Sheng et al., 2015). Chlorophyll level is a notable indicator of photosynthetic capacity (Kumar et al., 2022). Our results revealed that, increasing salinity levels significantly reduced Chl content in sweet pepper. The negative impacts of salinity stress on *C. annuum* including vegitative growth, chlorophyl content and crop yield reduction in addition to electrolyte leakage and proline, MDA and ROS accumulation was recorded by other studies (Abdelaal et al., 2020; Bello et al., 2021). Chlorophyll reduction under salinity stress could be explicated by the stimulated-pigment breakdown and surplus production of ROS (Choudhary et al., 2023). The restorative effect of SNPs on photosynthetic pigments was ascribed to their interaction with the other organic molecules forming organo-sulfur compounds, inducing chlorophyll biosynthesis by the leaves (Salem et al., 2016). Furthermore, Ragab and Saad-Allah (2020) confirmed that SNPs sustained a high level of Mg in the stressed plants lessening the deleterious impact on chlorophyll biosynthesis. Electrolyte leakage can be used as a useful damage indicator to detect the rate of injury in cell membrane under salt stress conditions, being the primary site of ion-specific damage due to salinity (Hniličková et al., 2019). Plants with low EL indexes are considered to be tolerant against stress. As clearly documented for several other plant species (Hatami et al., 2018; Mahlooji et al., 2018; Hniličková et al., 2019), current findings revealed that salinity increased EL from plasma membranes (as concluded from CMS% in **Figure 4B**), but leakage was attenuated with lower concentrations of SNPs treatments.

Relative water content (RWC), water potential (WP), solutes potential (SP) are important indexes for salinity tolerance, that describe the water status of plants that reflect the metabolic activity of tissues (Abdelmoghny et al., 2020). In this study, leaves RWC decreased significantly with increasing salt concentration, while the WP and SP depicted a significant rise with increasing salinity levels. Upon SNPs treatments, the RWC manifested a significant increase with increasing levels of SNPs while, the WP and SP decreased in sweet pepper leaves. It is reported that high RWC is a water stress tolerance mechanism and that high RWC is the result of more osmotic regulation or less elasticity of the cell wall (Zhang et al., 2021). Overall, our findings reveal osmotic adjustment due to seed priming with SNPs in sweet pepper leaves under salt stress. Lu et al. (2019) declared that SNPs restore water balance in the stressed plants allowing them to avoid osmotic and ionic imbalances leading to improved plant growth. Osmotic adjustment helps to increase the concentration of cellular solutes, which helps in maintaining the required osmotic potential that ensures continued uptake of water during the stress period (Blum, 2017).

Proline and glycine betaine (GB) are the main osmolytes that help to protect the integrity of the cell membrane and osmotic adjustment during water stress conditions (Kumar et al., 2011). Proline also contributes to stabilizing the subcellular structures (e.g., membranes and proteins), scavenges ROS, and buffers cellular redox potential under stress conditions (Zahid et al., 2021). Proline and GB concentration in sweet pepper leaves markedly increased by increasing salt concentration in this study. Controversially, SNPs decreased proline and GB accumulation in salt-stressed sweet pepper. This effect could be ascribed to the additional osmoprotectant and antioxidant potentials offered by SNPs, hence there is no need for further proline production in the presence of SNPs. The elevated Na^+^/K^+^ ratio due to salinity stress that observed in this study has many deleterious effects on plants. K^+^ retention in plant cells is known as a mechanism to improve salinity stress tolerance in crops (Ismail and Horie, 2017). Previous results showed that NPs assist in the higher retention of K^+^ in leaf mesophyll, resulting in improving plant photosynthetic performance under salinity stress (Boghossian et al., 2013). Our results showed similar findings, where application of SNPs enhanced Na^+^/K^+^ ratio in sweet pepper leaves under saline conditions.

The loss of membrane integrity and the increase in H_2_O_2_ and MDA concentrations as a common response to salt stress regarded as markers of oxidative stress. In the current study, MDA (as a measure of lipid peroxidation) and H_2_O_2_ and O_2_^-1^ (as measures of oxidative stress) were significantly increased in the leaves of sweet pepper exposed to salt stress. Similar results have been reported in various crop species (Sen et al., 2020; Shafiq et al., 2020). The substantial accumulation of MDA and H_2_O_2_ upon exposure to salinity was explained by the inadequate detoxification and antioxidant systems offered by the plant (Hossain et al., 2011). Likewise, the induced accumulation of MDA, H_2_O_2_ and O_2_^-1^ could be explained by the deficiency in the non-enzymatic antioxidants and the increasing cascade of ROS upon salt exposure. Our study demonstrates that seed priming with SNPs disclose a discernible recovery impact and lessened the stress biomarkers level in salt-stressed sweet pepper leaves. Similar results recorded in lettuce under salinity stress when treated with SNPs (Najafi et al., 2020).

The results of this study, showed that the activity of all the three antioxidant enzymes (SOD, APX, and CAT) increased significantly under salinity stress. Furthermore, this increase in the enzymes activity was more conspicuous and significant in response to SNPs treatments both under saline and non-saline conditions in sweet pepper leaves. Supporting to our results, recently, Khalifa et al. (2024) find that foliar treatments with SNPs, significantly enhanced antioxidant enzymes activities compared to salt-stressed faba bean. Enhanced antioxidant enzymes activity enables plants to eliminate the toxic levels of ROS and lower lipid peroxidation of cellular membranes under salinity. Increased SOD activity as a consequence of salt stress was attributed to the accelerating dismutation of superoxide anions generated upon salt-treatment. Moreover, the induction of CAT and POD activities was ascribed to their role in detoxifying H_2_O_2_ (Desingh and Kanagaraj, 2007). The increased activity of antioxidant enzymes has been ascribed to either increased activity of already present enzymes or upregulation of genes encoding these enzymes (Gupta and Gupta, 2005), which supported by the results of gene expression analysis in our study (**Figures 8G–I**).

The gene expression analysis provided valuable insights into the molecular mechanisms underlying alleviating salt stress in sweet pepper by SNPs seed priming (**Figures 8A–I**). HKT family genes belong to Trk superfamily which comprises a similar topology as K^+^ channels (Gu et al., 2023). In the current study, the influx of K^+^ regulating genes, *CaHAK6* and *CaHAK7* significantly upregulated in response to SNPs reaching their maximum levels at 10 mg/l under mild saline condition (50 mM NaCl). The upregulation of *CaHAK6* and *CaHAK7* was consistent with the influx K^+^ at corresponding concentrations of salinity and SNPs as indicated from **Figure 4C**, where Na^+^/K^+^ ratio reduced due to the increased influx K^+^. In addition, seed priming with SNPs significantly upregulated the expression level of proline-related gene, *CaPRP1*, compared to untreated plants under saline conditions. Although the exact physiological function of *CaPRP1* is not yet clear, there are several possibilities for its role in cell expansion and elongation during early development of hot pepper plants (Chawla et al., 2013).

In the current study, proline content varied inconsistently with the expression of *CaPRP1* under corresponding salinity treatments and SNPs in sweet pepper leaves which needs further studies. In additions, the ROS scavenging gene *CaDHN3* recorded maximum relative expression at 10 mg/l SNPs at all salinity concentrations. The expression of *CaDHN3* was consistent with the scavenging of H_2_O_2_ and O_2_^-1^ at corresponding treatments of salinity and SNPs. In the same context, Meng et al. (2021) reported that, *CaDHN3* play an important role in regulating the relative osmotic stress responses in plants through the ROS signaling pathway. Our study proved that that the overexpression of *CaDHN3* in sweet pepper impeded ROS damage under salt by decreasing MDA and EL% levels and enhancing POD, SOD, CAT and APX activities. *CaBiP1* and *CaBiP2* impart tolerance to stress by reducing MDA and ROS accumulation, and increasing the relative water content. Interestingly these genes showed maximum relative expression in sweet pepper leaves at higher SNPs treatments at all concentrations of salinity. The expression of *CaBiP1* and *CaBiP2* was more consistent with the accumulation of MDA at corresponding applications of treatments. In the same context, Wang et al. (2017) revealed that *CaBiP1* may contribute to tolerance to abiotic stress in pepper by reducing ROS accumulation, increasing the water retention ability, and enhancing the UPR pathways and expression of stress-related genes. Finally, the upregulation of antioxidant-related genes, such as *CaSOD*, *CaCAT1* and *CaPOD*, in response to SNPs treatments mainly under high stress condition (100 mM NaCl), underscores their crucial role in mitigating oxidative stress in sweet pepper. The expression of *CaSOD*, *CaCAT1 and CaPOD* in was in parallel with the enzymatic activities of CAT and POD.

Plant tolerance to stress factors is a highly complex process that involves the interactions of many component factors, known as contributing components. Thus, understanding the interaction of characters among themselves and with the environment is of crucial importance in plant physiology. In the present study, the strong positive correlations among physiological and biochemical traits such as Na^+^/K^+^, WP, and SP, osmolytes (GB and proline), and the activity of antioxidant enzymes (SOD, POD, CAT) under the effect of both treatments (salinity x SNPs), suggest a coordinated response to maintain water status, solute balance, and antioxidant system which are crucial under salinity stress. These traits are central to the plant’s ability to uphold cellular integrity and function during adverse environmental conditions (Li et al., 2019). Contrariwise, the negative correlations observed between physiological parameters like Chl and CMS indicate a disruption in cellular and photosynthetic efficiency due to ionic imbalances and oxidative stress (Ran et al., 2022). Indeed, the results from correlation analysis were further rectified by the PCA analysis and heat maps. Etesami et al. (2021) reviewed similar results for other NPs where they reported that, NPs applications lead to various profound effects on morphological, physiological, biochemical and molecular properties of plant various species. Overall, complex mechanisms and crosstalk between different biochemical cellular processes take place in the plant to cope with salt stress (Hajihashemi et al., 2020).

According to our findings, applying SNPs as seed priming treatment was notably successful in minimizing the impacts of salt hazards on growth, physio-chemical and molecular traits. According to a previous study, plant stresses like salinity might be alleviated by exogenous exposure to sulfur as a mineral additive (de Andrade et al., 2018). This is consistent with the findings of SNPs treatments on the growth of *Solanum lycopersicum* (Salem et al., 2016) and *Vicia faba* (Khalifa et al., 2024). Furthermore, in addition to the potted plant experiments carried out under controlled conditions, follow-up field trials could contribute to the understanding of potential applications of these advanced nanoparticles in agriculture under ever-changing environmental conditions, at the same time examining potential memory/trans-generational effects that may occur due to possible epigenetic modifications. In agriculture sector low yield under abiotic stresses is significant issue which is under consideration, therefore researchers should work in this field to explore the efficacy of SNPs in plant growth and agriculture under stress conditions. The impacts of ecofriendly and bio-based SNPs should be tested on different plants and agriculture crops. Further research work is needed to overcome the problems of low yield and control plants stress factors by the utilization of green synthesized SNPs and other important elements.

## 5 Conclusion

In conclusion, a novel, eco-friendly, rapid, and inexpensive method was used to synthesize SNPs using *M. oleifera* leaf extract. The findings of this study highlight also the potential of green SNPs as ecofriendly nano-fertilizers for mitigating the adverse effects of salt stress on growth and physiology of sweet pepper plants. Seed priming with SNPs enhanced growth parameters, osmotic adjustment, antioxidant defense system and gene expression levels in sweet pepper under salts stress, which highlight the multifaceted mechanisms underlying the stress-mitigating effects of SNPs. SNPs benefit the growth of sweet pepper because of their nutritional value, which is produced in plants through the synthesis of sulfur-containing biomolecules. Overall, a dual-functional SNPs as a nano-fertilizer that encourages plant growth while improving salinity tolerance in sweet pepper offers promising opportunities for the enhancement of sweet pepper’s productivity in salt affected regions. Hence, future studies should emphasis on adjusting the method of application and the effective concentrations of SNPs for diverse crops and soil conditions to additional improve their effectiveness in alleviating salt stress in addition to other abiotic stress conditions.

## Data Availability

The original contributions presented in the study are included in the article/**Supplementary Material**. Further inquiries can be directed to the corresponding author.
