# Supplementary material for: Green biogenic sulfur nanoparticles enhance Capsicum annuum (L.) resilience to salt stress by triggering physio-biochemical and genetic repair mechanisms
Source: Front Plant Sci. 2025 Mar 7;16:1564621. doi: 10.3389/fpls.2025.1564621 (PMC11925878; doi:10.3389/fpls.2025.1564621)
Supplement: Supplementary file 1 [file DataSheet1.pdf]

Green Biogenic Sulfur Nanoparticles Enhance *Capsicum annuum* (L.) Resilience to Salt Stress by Triggering Physio-biochemical and Genetic Repair Mechanisms

Hissah Alrabie[1], Hameed Alsamadany[1], Ameina S. Almoshadak[1], Rahma Alshamrani[1], Manal El-Zohri[1,2*]

Supplementary Figures

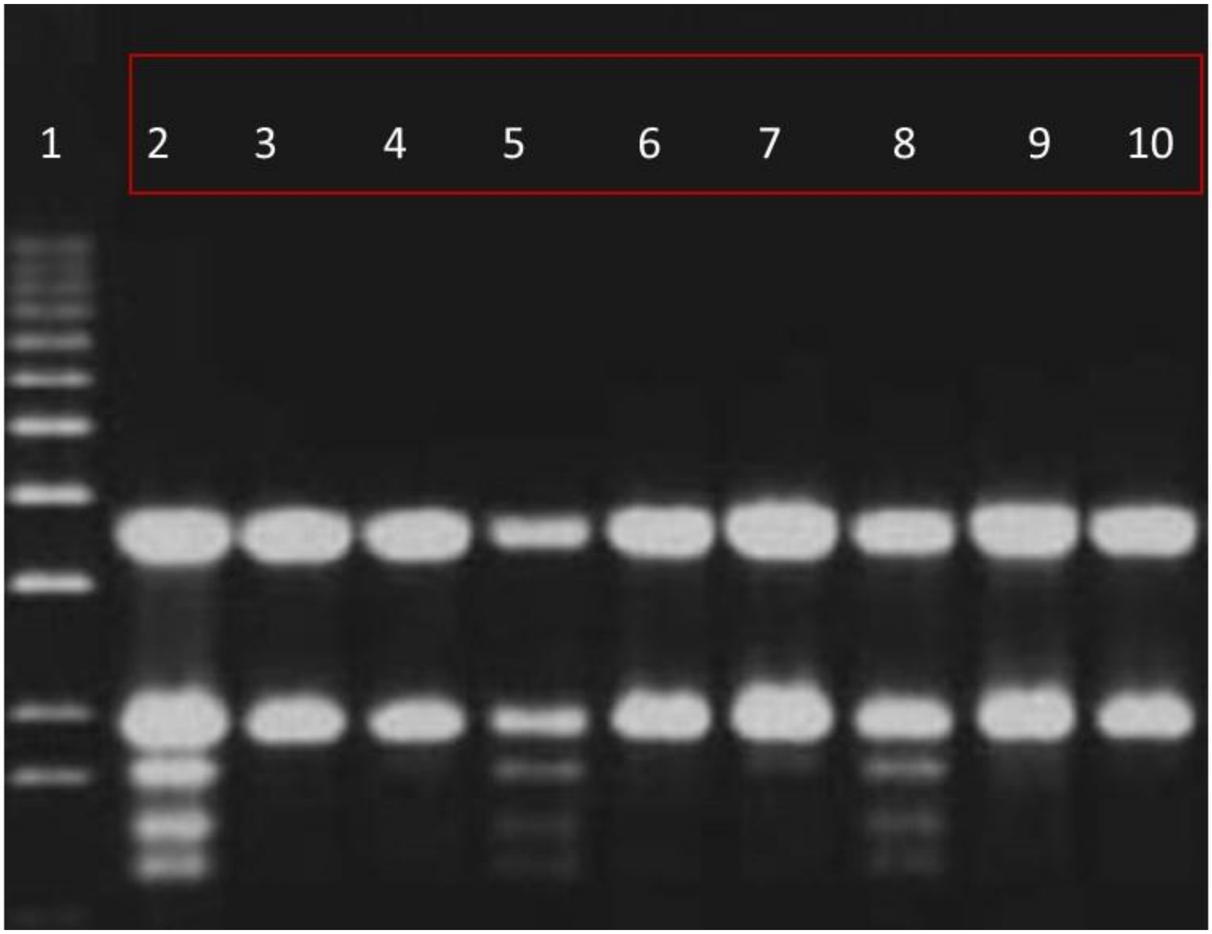

Figure S1. The electrophoresis gel demonstrating the quality of the extracted RNA

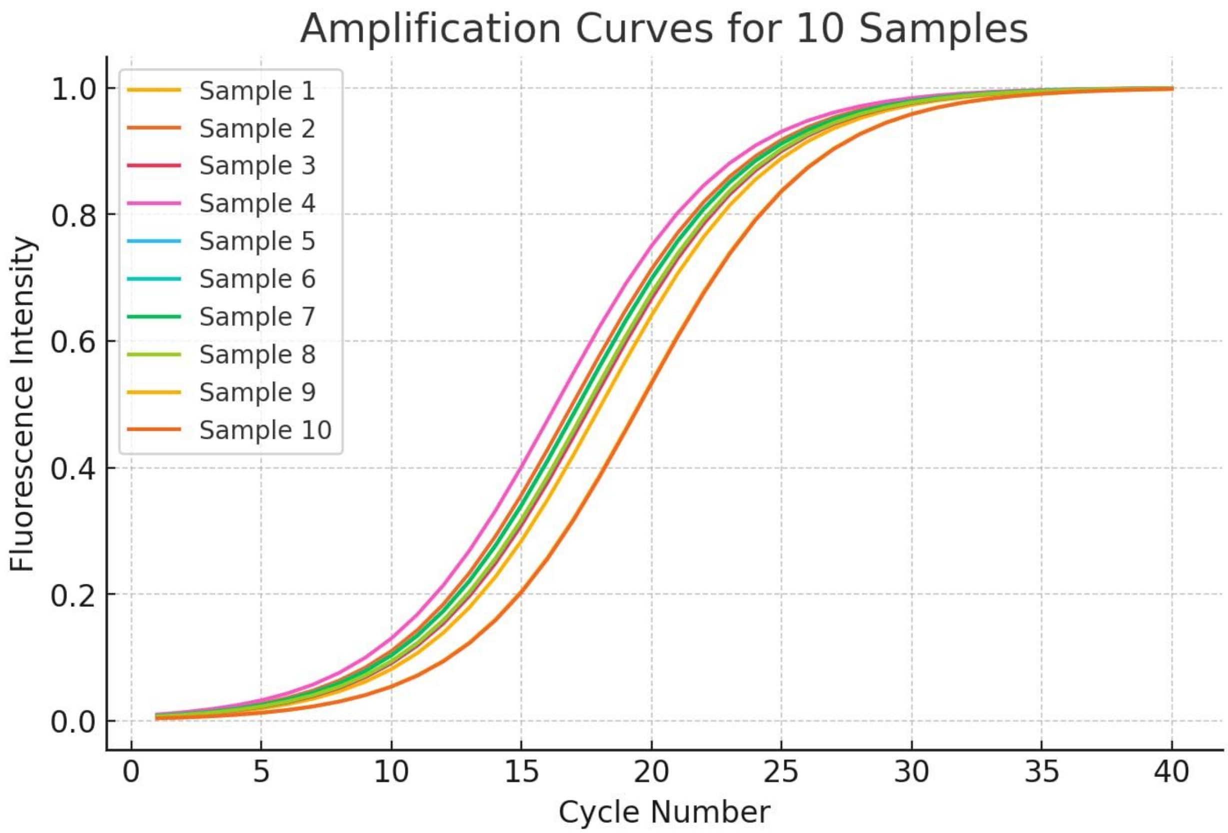

Figure S2: The amplification curves of the analysed genes

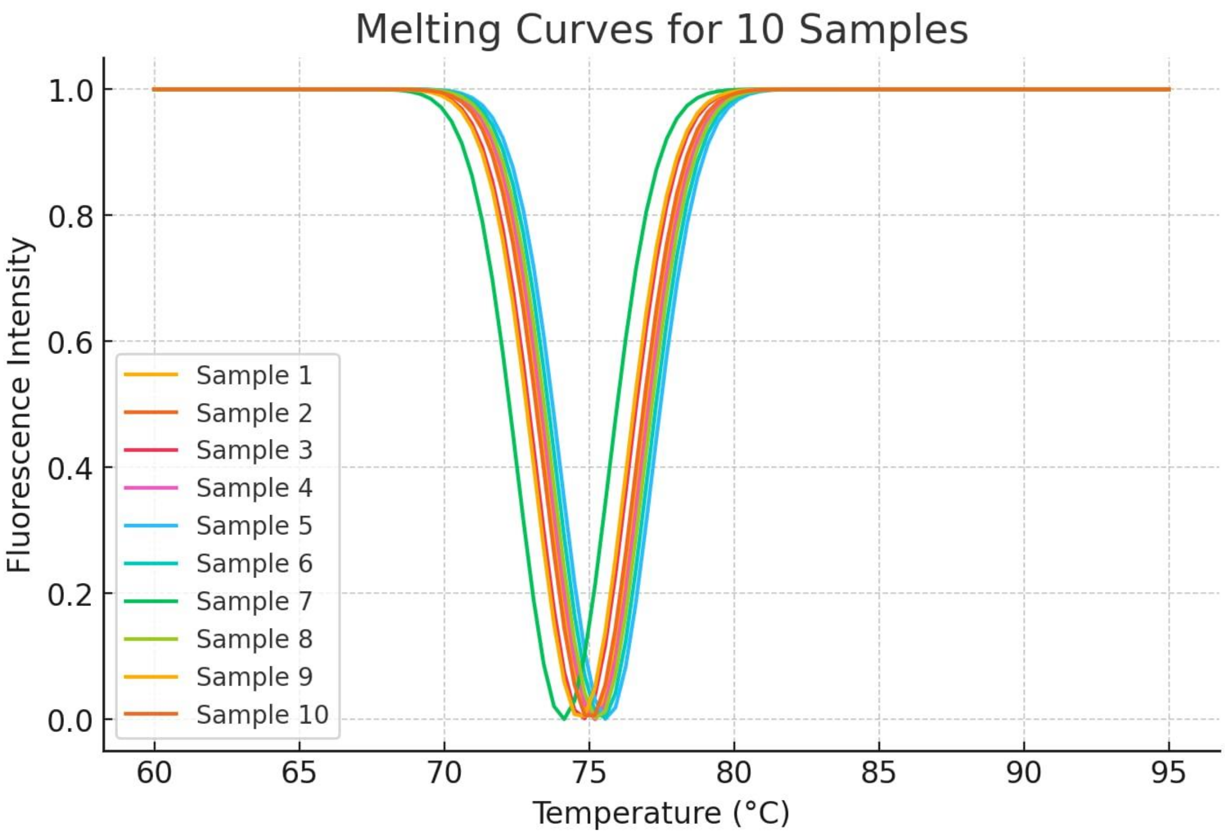

Figure S3: The melting Curves of the analysed genes
